# Effect of ischemic preconditioning on the expression of Cx43 protein in intestinal ischemia-reperfusion injury in SD rats

**DOI:** 10.3389/fphys.2026.1837072

**Published:** 2026-07-09

**Authors:** Ning Liu, Yibo Ma, Zhenzhong Han, Qian Zhang, Jianfeng Cao

**Affiliations:** 1 Department of Gastroenterology and Endocrinology, Xi ‘an North Hospital, Xi ‘an, China; 2 Department of Urology, Xi ‘an University of Science and Technology, Xi ‘an, China; 3 Xi ‘an Ninth Hospital, Xi ‘an, China

**Keywords:** ischemic preconditioning, intestinal ischemia-reperfusion injury, Connexin 43 (Cx43), oxidative inflammation, intestinal epithelial apoptosis

## Abstract

**Background:**

Intestinal ischemia reperfusion(I/R) injury is a life-threatening clinical condition. Ischemic preconditioning(IP) can alleviate I/R injury, but its intestinal protective mechanism is still unclear. Connexin 43 (Cx43) is involved in the pathological process of intestinal I/R. This study investigated the correlation between IP protection and Cx43 expression.

**Methods:**

32 male SD rats were randomly divided into normal group, sham operation group, I/R group and IP + I/R group. The normal group provided baseline data, and the sham operation group excluded surgical interference. The I/R model was established by clamping the superior mesenteric artery for 45 min and reperfusion for 2 h. IP was intervened before I/R modeling. MPO activity and Chiu’s score were detected. The location and level of Cx43 expression in small intestine tissue were observed by immunofluorescence detection and Western blotting. TUNEL staining was used to detect apoptosis in small intestine tissue.

**Results:**

There was no difference between the normal group and the sham operation group. Compared with the normal group, the level of MPO in I/R group was significantly increased (P<0.001), Chiu ‘s score was significantly increased (P<0.001), the expression of Cx43 protein in small intestine was significantly increased (P<0.001), and TUNEL staining positive cells were significantly increased (P<0.001). Compared with I/R group, the level of MPO in IP+I/R group was significantly decreased (p<0.001), Chiu ‘s score was significantly decreased (P<0.001), Cx43 protein expression was significantly decreased (P<0.001), and TUNEL staining positive cells were significantly decreased (P<0.001).

**Conclusion:**

Ischemic preconditioning can reduce intestinal I/R injury, which is related to the down-regulation of Cx43 expression. Cx43 may be a potential protective target.

## Introduction

Intestinal ischemia-reperfusion injury (IIRI) is a common life-threatening pathological condition, which is often secondary to mesenteric vascular occlusion, intestinal obstruction, shock and major abdominal surgery (Cai et al., 2022). This injury can induce intestinal mucosal necrosis, systemic inflammatory response syndrome, and even multiple organ dysfunction syndrome, with high mortality and lack of effective treatment (Deng et al., 2026; Marton et al., 2026). Therefore, it is of great clinical significance to elucidate the protective mechanism of IIRI and find potential therapeutic targets.

Ischemic preconditioning (IP) is an endogenous protective phenomenon that enhances tissue tolerance to subsequent long-term ischemia through transient and reversible ischemic stimulation (Riksen et al., 2024). IP has a clear protective effect on ischemia-reperfusion (I/R) injury of multiple organs such as heart, liver, kidney and brain (Ambros et al., 2007; Kim et al., 2021; Mouratidou et al., 2026). However, the molecular mechanism by which it reduces intestinal I/R injury has not been fully elucidated.

Connexin 43 (Cx43) is the most abundant gap junction protein expressed in intestinal epithelial cells and endothelial cells. It maintains tissue homeostasis by regulating intercellular transmission of ions, reactive oxygen species and inflammatory signals (Zheng et al., 2024; Zhu et al., 2024). Studies have shown that the expression of Cx43 is significantly up-regulated in intestinal I/R injury, which can promote oxidative stress, inflammatory infiltration and apoptosis, and then aggravate the destruction of intestinal mucosal barrier (Wang et al., 2023). However, it is not clear whether IP plays an intestinal protective role by regulating Cx43 expression, which is the core scientific problem to be solved in this study.

The purpose of this study was to establish a model of intestinal I/R injury in SD rats, to observe the effects of ischemic preconditioning on intestinal tissue injury, inflammatory response, Cx43 expression and apoptosis, and to explore the potential role of Cx43 in IP-mediated intestinal protection. The results are expected to provide new theoretical basis and potential therapeutic targets for the clinical prevention and treatment of IIRI.

## 1 Materials and methods

### 1.1 Experimental animals

Thirty-two healthy male SD rats, aged 10–13 weeks, weighing 250 ± 30 g, were purchased from the Experimental Animal Center of Xi ‘an Jiaotong University (License No.: SCXK shan 2023-002). All animal experiments were approved by the Ethics Committee of Xi ‘an North Hospital (Ethical Batch No.: 2024011) and strictly followed the guidelines for the feeding and use of experimental animals. Rats were fed in a specific pathogen-free (SPF) animal room, 4 rats per cage, ambient temperature 24 ± 2 °C, humidity 50 ± 5%, free access to food and water.

### 1.2 Main reagents and instruments

Rabbit anti-Cx43 polyclonal antibody, mouse anti-β-actin monoclonal antibody, goat anti-rabbit IgG secondary antibody, goat anti-mouse IgG secondary antibody and ECL chemiluminescence substrate solution were purchased from Wuhan Boster Biological Engineering Co., Ltd.; TUNEL BrightGreen apoptosis detection kit was purchased from Nanjing Novizan Biotechnology Co., Ltd.; DAPI staining reagent was purchased from Shanghai Biyuntian Biotechnology Co., Ltd. The main instruments include: fluorescence microscope (Olympus), X-ray film (Ruike Medical Equipment Co., Ltd.), development and fixing kit (Tianjin Century Aobo Commerce and Trade Co., Ltd.), high-speed frozen centrifuge (Hunan Kecheng), microplate reader (Shenzhen Huisong), electrophoresis instrument (Beijing Liuyi Biotechnology Co., Ltd.), high-speed centrifuge (Germany Eppendorf) and 69001/ThermoStar body temperature maintenance instrument (Shenzhen Rivard Biotechnology Co,Ltd.).

### 1.3 Method

#### 1.3.1 Establishment of intestinal ischemia-reperfusion injury model in male SD rats

Thirty-two healthy male SD rats were divided into 4 groups by computer random number table method, 8 rats in each group, namely normal group (Normal group), sham operation group (Sham group), intestinal ischemia-reperfusion group (I/R) and ischemic preconditioning combined with ischemia-reperfusion group (IP + I/R). The rats were fasted for 24 h and forbidden to drink for 4 h before operation, and were anesthetized by intraperitoneal injection of 50 mg/kg 3% pentobarbital sodium. Under sterile conditions, the rats were fixed on their backs and the abdominal skin preparation and disinfection were completed. During the operation and resuscitation stage, the rectal temperature was continuously monitored by 69001/ThermoStar temperature maintenance instrument, and the body temperature of the rats was maintained at 37.0 ± 0.5 °C. After surgery, they were kept in a constant temperature cage at 28-30 °C until they were fully awakened. In this study, the ischemic preconditioning model was constructed according to the published classical modeling scheme at home and abroad (Vlasov et al., 2002; Gonzalez et al., 2015; Wang et al., 2021; Pan et al., 2022; Henein et al., 2023). The pretreatment intervention parameters and ischemia-reperfusion time course were set according to the mature experimental system in the field of ischemia-reperfusion injury (Vlasov et al., 2002; Wong et al., 2021). Normal group: no surgical operation after anesthesia; sham group: only laparotomy was performed to expose the free superior mesenteric artery (SMA) without clipping, and the abdomen was closed for 2 h. In the I/R group, the superior mesenteric artery was clipped for 45 min followed by reperfusion for 2 h; the IP + I/R group was given 10 min ischemia and 10 min reperfusion pretreatment, followed by the same duration of ischemia-reperfusion treatment. After the experiment, high-dose pentobarbital sodium (150mg/kg) was used to euthanize according to animal ethical norms, reduce experimental stress, ensure stable expression of Cx43 protein, and adapt to subsequent experimental tests. This method meets the requirements of AVMA, CCAC and Chinese national standard GB/T 39760-2021. The ethics committee of Xi ‘an North Hospital approved all euthanasia procedures (approval number: 2024011).

#### 1.3.2 The activity of myeloperoxidase (MPO) in small intestine of rats in each group was detected

The small intestinal tissue of each group was placed in a 5mL beaker and made into 10% tissue homogenate with a homogenizer. The MPO activity was detected by visible spectrophotometry. The specific method was based on the instructions of the myeloperoxidase (MPO) activity detection kit (Beijing Soleibao Technology Co., Ltd.).

#### 1.3.3 Pathological examination of small intestine tissue and Chiu ‘s pathological score were performed in each group

After the rats were sacrificed at each preset time point, the small intestine tissue about 10 cm from the proximal end of the ileocecal region was immediately intercepted, and the intestinal contents were removed by washing with sterile saline. The specimens were divided into two parts, one was fixed in 4% neutral buffered formaldehyde solution for histopathological analysis; the other was cryopreserved at -80 °C for subsequent Western blot analysis. The fixed tissues were embedded in paraffin, and 5 μm serial sections were made and stained with hematoxylin-eosin (HE). The tissue morphology was observed by optical microscope and the images were collected. The Chiu scoring system was used to evaluate the degree of intestinal mucosal injury. In order to avoid observation bias, all pathological assessments were performed independently by two pathologists who were unaware of the experimental grouping. All slices are randomly coded, and the grouping information is unlocked after all scoring work is completed. The final score of each sample was taken as the average of the independent scores of the two researchers. If the score difference exceeded 1 point, the senior pathologist would review and decide to achieve a unified result. Chiu ‘s scoring criteria are as follows (Quaedackers et al., 2000): 0 points, small intestinal villus morphology is normal; 1, the top of the villi formed a subepithelial space, accompanied by telangiectasia; 2 points, the subepithelial space was enlarged, and the lamina propria was moderately edematous; 3, epithelial cell degeneration and necrosis, lamina propria edema was significant; 4 points, villous necrosis and shedding, vasodilation, and increased inflammatory infiltration of lamina propria; 5 points, lamina propria structure disintegration, vascular bleeding, and intestinal ulcer.

#### 1.3.4 The expression of Cx43 protein was detected by immunofluorescence

Paraffin sections with a thickness of 4μm were prepared. The slices were dewaxed and hydrated with xylene and a series of gradient ethanol solutions, and then washed three times with distilled water. After antigen repair, the sections were washed three times with PBS. The sections were incubated overnight at 4 °C with primary antibody, and rewarmed before adding secondary antibody. Subsequently, DAPI re-staining was performed and sealed with a cover glass. Observed under a fluorescence microscope. The average fluorescence intensity of the stained sections was detected using Image J computer software.

#### 1.3.5 The expression of Cx43 protein was detected by Western blotting

The frozen intestinal tissue was added to the protein lysis buffer to grind and extract the protein. After centrifugation, the supernatant was separated and the protein concentration was determined using the BCA kit and balanced. 15 μg of samples from each group were added to gel electrophoresis, transferred to PVDF, and blocked for 2 h. TBST was rinsed three times and incubated with Cx43 primary antibody (1: 1000) at 4 °C overnight. TBST was rinsed three times on the next day, and Cx43 secondary antibody (1: 10000) was added to incubate for 2 hours. The color was exposed in the dark room and fixed after development. The gray value of protein bands was measured by ImageJ, and the gray ratio of Cx43 to β-actin was calculated by using β-actin protein as an internal reference for semi-quantitative analysis.

#### 1.3.6 TUNEL (fluorescence) staining

TUNEL staining was used to detect the apoptosis of small intestinal tissue. The paraffin sections of small intestinal tissue were dewaxed and hydrated. The prepared protease K working droplets were added to the sections, placed in a wet box and incubated in a 37 °C incubator for 20 min. After the end of the experiment, phosphate buffered saline (PBS) was used to clean the sections, and TUNEL detection solution was added to the sections to completely cover the sample tissues, and the samples were continuously incubated in a wet box for 1 h in the dark. The excess staining solution was washed with PBS, and then the DAPI staining solution was evenly added to the slices, and the nuclei were stained in dark at room temperature for 10 min. PBS was washed again, sealed with anti-fluorescence quencher, observed by fluorescence microscope and collected images. Three non-overlapping fields of vision were randomly selected from each slice, and the average fluorescence intensity of apoptotic cells was detected by computer software ImageJ.

### 1.4 Statistical analysis

In this study, SPSS 25.0 software was used for data analysis, and the measurement data were expressed as mean ± standard deviation. Before statistical analysis of all data, Shapiro-Wilk test was used for normality test and Levene test for homogeneity of variance test. For data with normal distribution and homogeneity of variance, one-way analysis of variance (ANOVA) was used for comparison between multiple groups, and independent sample t test was used for comparison between two groups. This study is a mechanism exploratory experiment, and the number of preset inter-group comparisons is limited. Therefore, the LSD method is used for ANOVA *post hoc* comparison. For data with uneven variance, Tamhane T2 test was used. All quantitative analysis results of this study reported accurate P values to improve statistical transparency, and P < 0.05 was considered statistically significant.

## 2 Result

### 2.1 The level of myeloperoxidase (MPO) in small intestine

**Figure 1:**
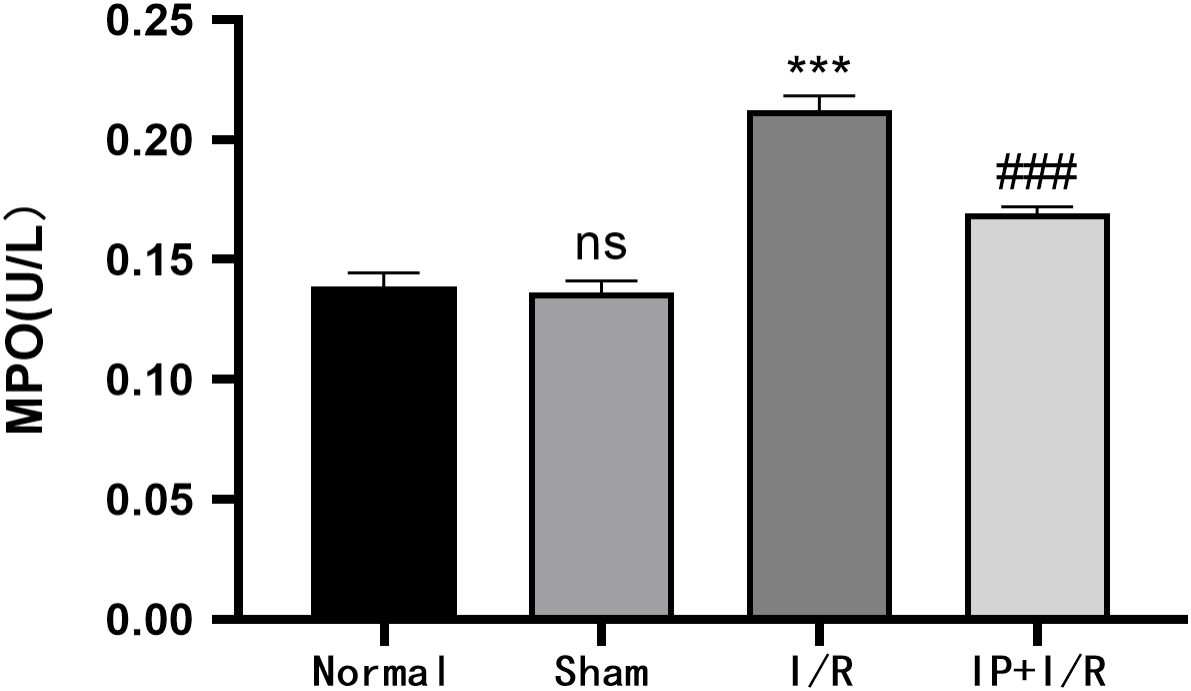
The level of MPO in small intestine tissue of rats in each group. One-way ANOVA and Tukey ‘s test were used for statistical analysis. The data were expressed as mean ± SEM. ***p < 0.001 vs Normal group, ###p < 0.001 vs I/R group. I/R, ischemia-reperfusion; IP, ischemic preconditioning.

As shown in **Figure 1**, there was no significant difference between the Normal group and the Sham group, and the MPO level in the I/R group was significantly higher than that in the Normal group (p < 0.001). The level of MPO in IP + I/R group was significantly lower than that in I/R group (p < 0.001).

### 2.2 HE staining of small intestine tissue

**Figure 2:**
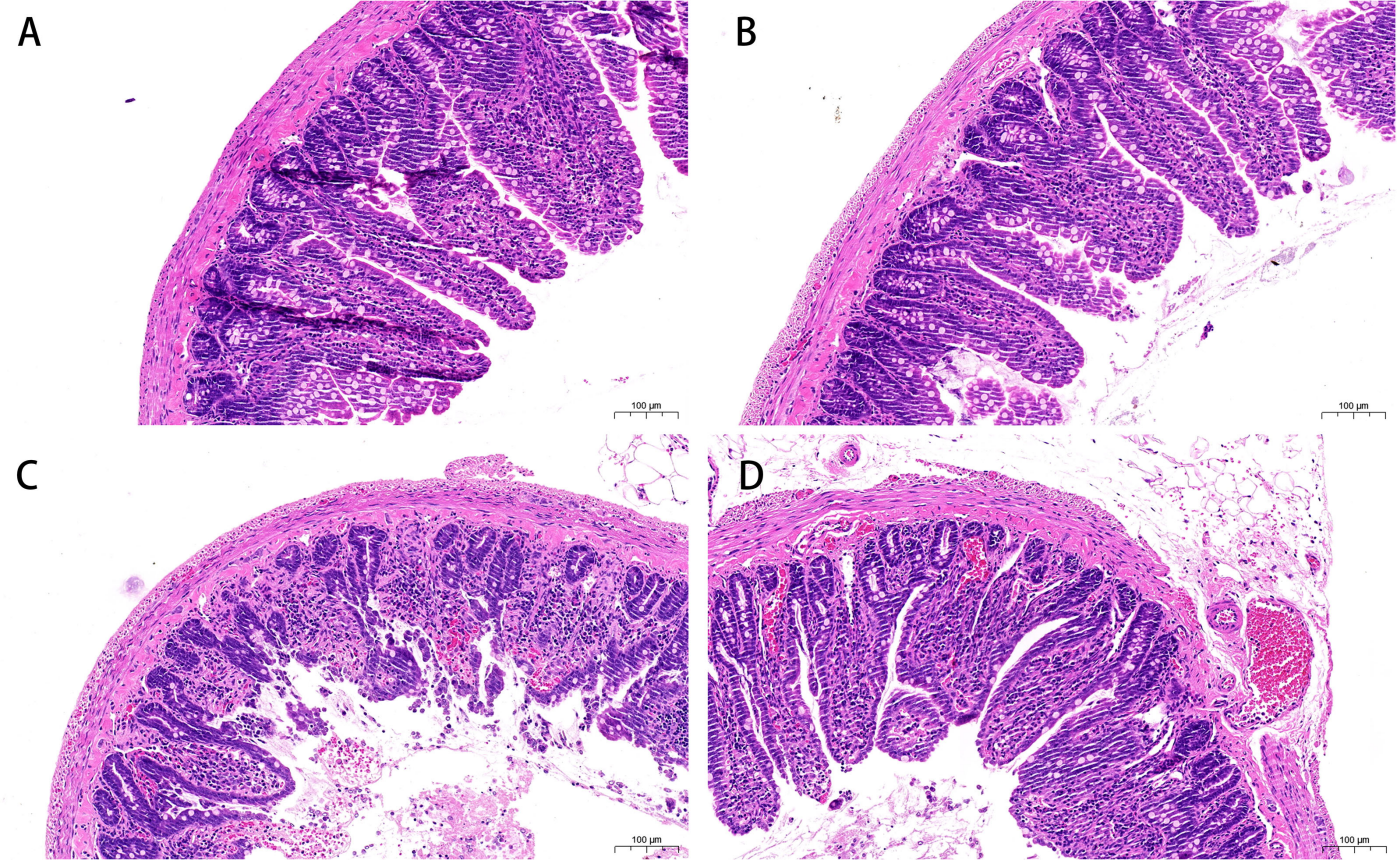
Pathological changes of small intestine tissue in each group (HE staining). **(A)** Normal group; **(B)** Sham group; **(C)** I/R group; **(D)** IP + I/R group. I/R: ischemia-reperfusion; IP: ischemic preconditioning.

As shown in **Figure 2**, the results of HE staining under CaseViewer after slice scanning showed that the morphological structure of the small intestinal mucosa in the Normal group was basically complete, the villi were arranged neatly, and the goblet cells were visible; in the Sham group, the morphology of intestinal mucosal villi was normal; in the I/R group, the intestinal mucosal tissue was severely damaged, which was manifested as edema of intestinal mucosal tissue, disappearance of tissue lamina propria, ulcer formation, disruption and disappearance of intestinal villus tissue, obvious congestion and damage of intestinal blood vessels. In the IP + I/R group, the intestinal mucosal villus tissue morphology was clear, and slight intestinal epithelial space expansion and intestinal mucosal epithelium elevation were observed, and the intestinal tissue damage was lighter.

### 2.3 Chiu ‘s score of small intestinal histopathology

As shown in **Table 1**, according to the Chiu ‘s scoring system, there was no significant difference between Normal group and Sham group (P > 0.05). The Chiu ‘s score of I/R group was significantly higher than that of Normal group (P < 0.001), and the Chiu ‘s score of IP + I/R group was significantly lower than that of I/R group (P < 0.001).

**Table 1:** Comparison of Chiu ‘s scores of small intestine pathology in rats of each group (x ± s).

Grouping	Score
Normal group	0.2 ± 0.426
Sham group	0.6 ± 0.426
I/R组	4.3 ± 0.725***
IP+I/R group	2.2 ± 0.617###

**Table 1**: Comparison of Chiu ‘s scores of small intestine pathology in each group. ANOVA was used for *post hoc* comparison and LSD was used for statistical analysis. The data were expressed as mean ± SEM. ***p < 0.001 vs Normal group, ###p < 0.001 vs I/R group. I/R, ischemia-reperfusion; IP, ischemic preconditioning.

### 2.4 The expression location of Cx43 protein in small intestine tissue

**Figure 3:**
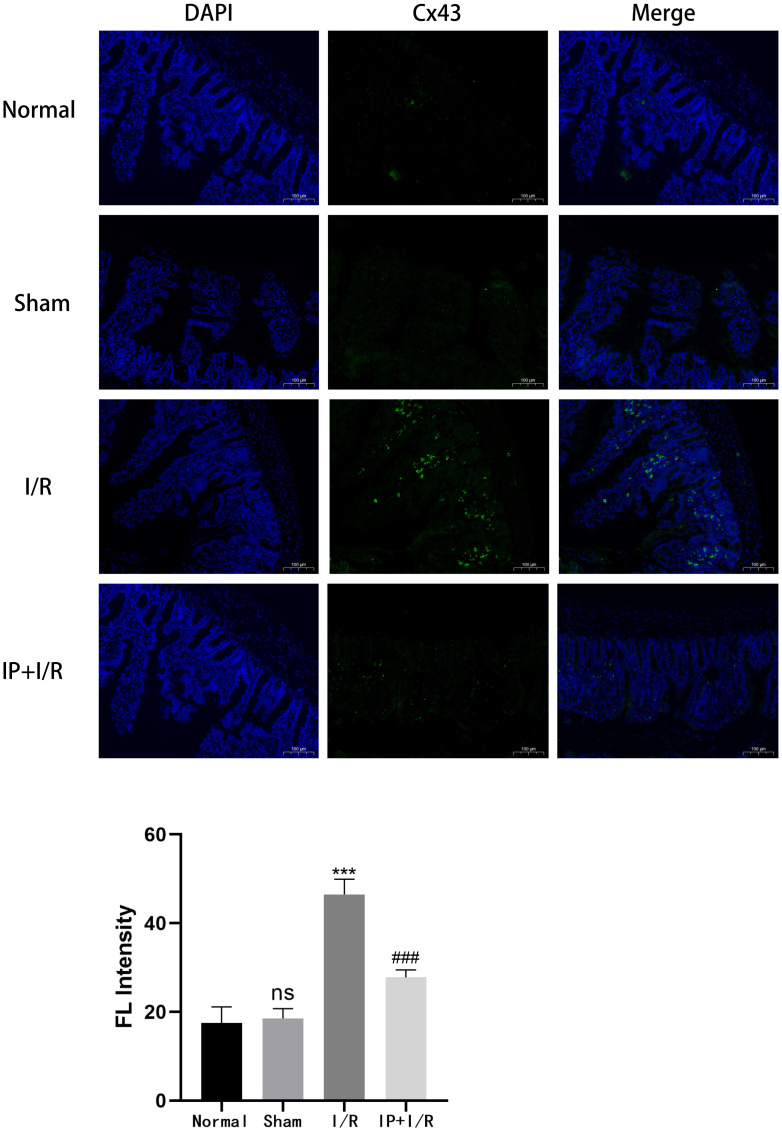
The expression location of Cx43 protein in small intestine of rats in each group (immunofluorescence). DAPI marked the nucleus. Green: Cx43. Bar: 100μm. One-way ANOVA and Tukey ‘s test were used for statistical analysis. The data were expressed as mean ± SEM. ***p < 0.001 vs Normal group, ###p < 0.001 vs I/R group. I/R, ischemia-reperfusion; IP, ischemic preconditioning.

As shown in **Figure 3**, the results of immunofluorescence showed that Cx43 protein was expressed in the cell membrane and cytoplasm in the small intestine. Because Cx43 protein is a gap junction protein, it is mainly distributed on the cell membrane. There was no significant difference between Normal group and Sham group (P > 0.05). The expression of Cx43 protein was significantly increased in the I / R group compared with the Normal group (P < 0.001), and the expression of Cx43 protein was significantly decreased in the IP + I / R group compared with the I / R group (P < 0.001).

### 2.5 Comparison of the expression levels of Cx43 protein

**Figure 4:**
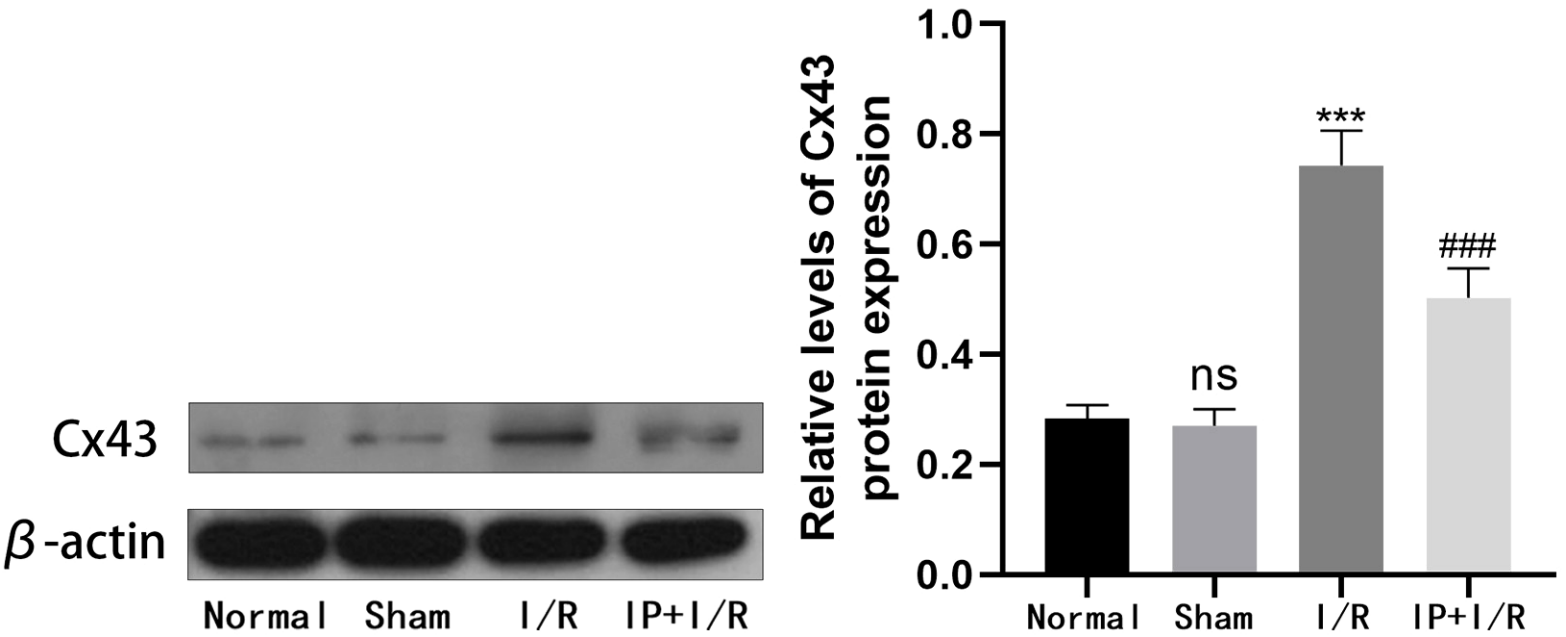
The expression of Cx43 in small intestine of rats in each group (Western blot). One-way ANOVA and Tukey ‘s test were used for statistical analysis. The data were expressed as mean ± SEM. *** p < 0.001 vs Normal group, ### p < 0.001 vs I/R group. I/R, ischemia-reperfusion; IP, ischemic preconditioning.

As shown in **Figure 4**, Western blot results showed that there was no significant difference between Normal group and Sham group (P > 0.05). The expression of Cx43 protein in I/R group was significantly higher than that in Normal group (P < 0.001), and the expression of Cx43 protein in IP + I/R group was significantly lower than that in I/R group (P <0.001).

### 2.6 TUNEL results

**Figure 5:**
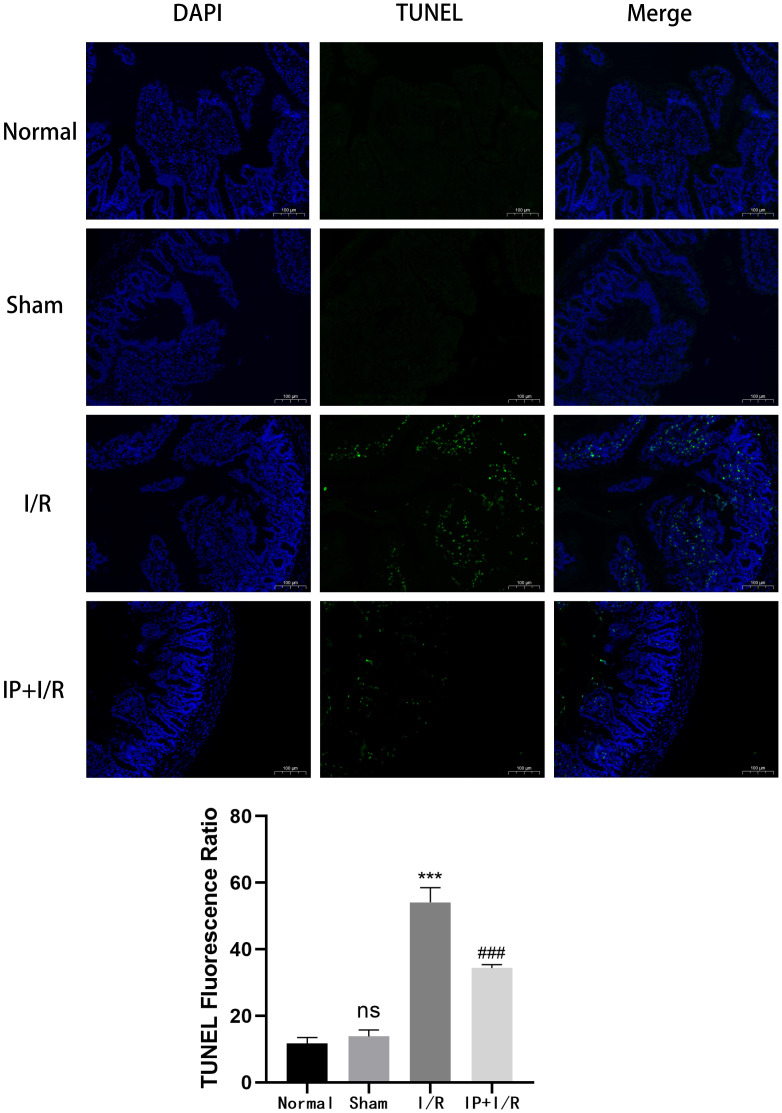
TUNEL was used to detect apoptosis in small intestine of rats in each group. DAPI marked the nucleus. Green: apoptotic cells. Bar: 100μm. One-way ANOVA and Tukey ‘s test were used for statistical analysis. The data were expressed as mean ± SEM. ***p < 0.001 vs Normal group, ###p < 0.001 vs I/R group. I/R, ischemia-reperfusion; IP, ischemic preconditioning.

As shown in **Figure 5**, TUNEL results showed that the positive expression of TUNEL staining in Normal group and Sham group was less; TUNEL staining positive cells were significantly increased in the I/R group compared with the Normal group (P < 0.001), and TUNEL staining positive cells were significantly decreased in the IP + I/R group compared with the I/R group (P < 0.001).

## 3 Discussion

IIRI is a common and serious pathological injury in the progression of various clinical critical diseases. The recovery of blood perfusion after transient ischemia of the small intestine will not repair tissue damage, but will induce oxidative stress, inflammatory cascade activation and excessive apoptosis of epithelial cells, which will further aggravate the damage of intestinal mucosal barrier. In severe cases, it can induce systemic inflammatory response and multiple organ dysfunction (Gordeeva et al., 2017; Deng et al., 2026; Marton et al., 2026). IP is an efficient endogenous protection strategy that can effectively antagonize multiple organ I/R injury by activating the body ‘s pre-adaptive defense mechanism (Moore-Olufemi et al., 2005; Khbouz et al., 2023; Tian et al., 2024; Zhang et al., 2026), but its specific molecular regulation mechanism in intestinal I/R injury has not been fully elucidated. Based on the previous pre-experimental results and reference literature (Gonzalez et al., 2015; Wang et al., 2021), this experiment chose to ligate the superior mesenteric artery for 45 min and reperfusion for 2h to establish a model, because the model can better simulate the pathological process of IIRI in clinical practice, and under this time parameter, the degree of injury is moderate, which is convenient for observing the intervention effect of ischemic preconditioning.

In the process of IIRI, a large number of neutrophils are activated and infiltrated into the intestinal tissue (Chu et al., 2023). As a marker of neutrophils, the increase of MPO activity or content usually reflects the degree of aggregation and activation of neutrophils in the intestinal tract, which indirectly reflects the intensity of inflammatory response (Cheng et al., 2024; Zhong et al., 2025; Deng et al., 2026; Yang et al., 2026). Studies have shown that MPO activity in intestinal tissue after IIRI will be significantly increased, and it is positively correlated with the degree of intestinal injury. MPO strongly catalyzes the production of a large amount of reactive oxygen species ROS, causing severe oxidative stress (Deng et al., 2026; Yang et al., 2026). IP reduces neutrophil adhesion, infiltration, and activation from the source by activating endogenous anti-inflammatory, endothelial protection, and chemokine inhibition pathways in advance, and ultimately directly down-regulates tissue MPO levels (Moore-Olufemi et al., 2005; Xie et al., 2025; Mouratidou et al., 2026). The results of this experiment showed that the MPO activity of I/R group was significantly higher than that of Normal group. The activity of MPO in IP + I/R group and I/R group decreased, suggesting that the protective effect of IP on IIRI was related to the reduction of intestinal inflammation and oxidative stress.

The Chiu ‘s score results of the pathological morphology of the small intestine showed that the rats in the I/R group showed that the lamina propria of the small intestine mucosa was hydrolyzed or even destroyed and disappeared, a large number of bleeding points and ulcer formation, inflammatory cell infiltration between tissues and accompanied by a large area of blood vessels were obviously congested. The Chiu ‘s score was significantly higher than that of the Normal group, which confirmed that IIRI had serious intestinal injury, which was consistent with the previous research results (Ji, 2015). At the same time, it also suggested that the rat intestinal ischemia-reperfusion injury model was successfully established. After using ischemic preconditioning rats before surgical ischemia, the morphological structure of intestinal mucosal villi in IP + I/R group was less damaged under the microscope, and mild congestion of capillaries was observed. The Chiu ‘s score of small intestine pathology also supported the above statement, suggesting the effectiveness of IP in reducing IIRI in rats after ischemia-reperfusion injury.

Connexin 43 (Cx43) is a core gap junction protein highly expressed in intestinal epithelial cells. It is mainly involved in intercellular signal transduction, material exchange and microenvironment homeostasis regulation. Its abnormal expression is closely related to intestinal oxidative stress disorder, inflammatory infiltration and apoptosis process (Wang et al., 2023; Zou et al., 2023; Liu et al., 2024). A large number of studies have confirmed that Cx43 plays a key role in ischemia-reperfusion (I/R) injury of important organs such as heart and brain. Its abnormal expression is closely related to the degree of tissue injury, inflammatory response and apoptosis, and is a common core molecule that mediates I/R injury of multiple organs. Marisol Ruiz-Meina et al.found (Ruiz-Meana et al., 2025) that the abnormal accumulation of ROS in myocardial cells during hypoxia induced the dephosphorylation of Cx43, which led to the depolymerization of the gap junction structure of the cell membrane. Subsequently, Cx43 on the surface of the membrane was endocytosed by the clathrin-mediated pathway, and a large number of Cx43 was transferred into the cytoplasm. The results of immunofluorescence and Western blot showed that the expression of Cx43 in I/R group was higher than that in Normal group, and mainly expressed in cell membrane and cytoplasm. It may be similar to the mechanism of Cx43 protein change in myocardial ischemia injury, which needs further experimental verification. Compared with I/R group, the expression of Cx43 in intestinal tissue of IP + I/R group decreased, and the down-regulation trend of Cx43 expression was highly consistent with the improvement of intestinal injury, the relief of inflammation and the decrease of apoptosis. It was confirmed that IP had a protective effect on intestinal I/R injury in rats, and was closely related to the inhibition of abnormal expression of Cx43 protein and the decrease of inflammation. According to Min Li et al.‘s study (Li et al., 2025), in the study of cerebral ischemia-reperfusion injury, ischemia-reperfusion injury can induce the expression of CX43 in astrocytes, make the gap junction and semi-channel over-open, promote the proliferation of inflammatory mediators, and aggravate inflammatory infiltration. Accumulated inflammatory factors further up-regulate the expression of CX43, forming a vicious cycle of CX43-inflammation positive feedback, and continuously magnifying the injury. Ischemic preconditioning can inhibit the abnormal high expression of CX43 induced by ischemia-reperfusion injury, block the vicious cycle of CX43-mediated ROS and inflammation positive feedback, reduce the diffusion of inflammatory mediators and the infiltration of inflammatory cells, and then reduce the intestinal ischemia-reperfusion injury by adapting the small intestinal tissue to hypoxic stress in advance and producing endogenous ischemic tolerance.

During IIRI, ischemia can lead to cell energy metabolism disorders, insufficient ATP production, and intracellular calcium overload, which in turn induces mitochondrial dysfunction and oxidative stress. After reperfusion, a large amount of reactive oxygen species (ROS) burst, further damaging cell membranes, DNA and proteins, and ultimately activating endogenous apoptotic signaling pathways to accelerate apoptosis of intestinal epithelial cells. In the study of Wang et al. (2025), the down-regulation of Cx43 expression, membrane distribution disorder and phosphorylation imbalance in cardiac I/R injury will aggravate the diffusion of injury signals between cardiomyocytes and amplify the apoptotic response; ischemic preconditioning can up-regulate the expression of functional Cx43, stabilize gap junction communication and significantly reduce the apoptosis rate of cardiomyocytes. Yingzhu Chen et al.showed (Chen et al., 2018) that in cerebral I/R injury, abnormal high expression of Cx43 in astrocytes mediates inflammation and excitatory toxic signal transduction, and promotes neuronal apoptosis; ischemic preconditioning can moderately inhibit the overexpression of Cx43, block the cascade transmission of apoptotic signals, reduce neuronal apoptosis and improve neurological function. Magali Genest et al (Genest et al., 2024).have shown that in renal I/R injury, the decrease of Cx43 expression in renal tubular epithelial cells leads to the decoupling of gap junctions, aggravates calcium overload and oxidative stress, and promotes apoptosis of renal tubular epithelial cells. Ischemic preconditioning can restore the normal expression and function of Cx43, reduce mitochondrial damage, inhibit the activation of apoptotic pathways, and reduce the apoptosis level of renal tubular epithelial cells. The results of TUNEL in this study showed that the number of apoptotic positive cells in the small intestine of the Normal group and the Sham group was very small, and the level of apoptosis was in the baseline state; compared with the Normal group, the number of apoptotic positive cells in the I/R group was significantly increased (P < 0.001), suggesting that I/R injury can significantly induce apoptosis of intestinal epithelial cells; the number of apoptotic cells in IP + I/R group was significantly lower than that in I/R group (P < 0.001), indicating that ischemic preconditioning can effectively inhibit intestinal I/R injury-mediated apoptosis, which is consistent with the anti-apoptotic protective effect of IP in previous studies of heart, brain, kidney and other organs, suggesting that Cx43 is a common key target for ischemic preconditioning to regulate apoptosis in multiple organ I/R injury.

The results of this study showed that there was no significant difference in intestinal pathological morphology, myeloperoxidase (MPO) activity, apoptosis level and Cx43 basic expression level between the normal group and the sham operation group. It was confirmed that simple anesthesia, abdominal laparotomy and superior mesenteric artery dissociation would not cause obvious intestinal injury, nor would they interfere with the basic expression level of Cx43. The control setting of this study was scientific and reasonable, which effectively excluded the interference of surgical trauma on the experimental results. At this stage, this study only verified the correlation between the two, and has not yet further verified the direct causal relationship through targeted interventions such as Cx43 specific inhibitors, gene knockdown or overexpression, and only detected the overall expression level of Cx43. The regulation of downstream NF-κB inflammatory pathway, ROS metabolism and gap junction function is not discussed in depth. In the future, it is still necessary to improve the molecular mechanism verification to clarify the core mediating role of Cx43. Combined with previous studies, it has been confirmed that reperfusion 2 h is the most significant time node for intestinal pathological damage. This study selected this time point to focus on the early protective effect of IP, which can objectively reflect the intervention effect of IP on acute intestinal I/R injury. This study did not dynamically monitor the changes of intestinal barrier function, intestinal motility and systemic inflammation at a longer time node, so it was not possible to fully reveal the continuity and dynamic evolution of IP protection effect.

In summary, this study confirmed that IP can effectively reduce intestinal I/R injury in rats through multi-dimensional experiments. Its protective effect is closely related to down-regulation of intestinal Cx43 protein expression, inhibition of inflammatory infiltration and apoptosis, and alleviation of oxidative stress injury. This study verified for the first time that IP could significantly reverse the abnormal high expression of Cx43 induced by intestinal I/R injury, and clarified the close relationship between Cx43 and intestinal I/R injury and IP protective effect, which provided new experimental clues and potential targets for the mechanism research and targeted intervention of intestinal I/R injury. Subsequent studies will further improve the verification of Cx43 targeted intervention, expand the observation time dimension and analyze the upstream and downstream signal regulation networks, improve the molecular mechanism of IP intestinal protection, and provide a solid theoretical basis and experimental support for clinical prevention and treatment of intestinal I/R injury.

## Data Availability

The datasets presented in this study can be found in online repositories. The names of the repository/repositories and accession number(s) can be found in the article/supplementary material.
